# Supplementary material for: Aberrant fragmentomic features of circulating cell-free mitochondrial DNA as novel biomarkers for multi-cancer detection
Source: EMBO Mol Med. 2024 Oct 30;16(12):3169–83. doi: 10.1038/s44321-024-00163-6 (PMC11628560; doi:10.1038/s44321-024-00163-6)
Supplement: Supplementary file 10 — Appendix [file 44321_2024_163_MOESM10_ESM.pdf]

Appendix

Table of contents:

Appendix Figure S1 | Reproducibility of ccf-mtDNA fragmentomic features among three replicated experiments (Rep1-3). 1

Appendix Figure S2 | Influence of sequencing depth on ccf-nDNA and ccf-mtDNA fragmentomic features. 3

Appendix Figure S3 | Different fragmentomic features between ccf-nDNA and ccf-mtDNA from patients with different disease types. 5

Appendix Figure S4 | Consistency of ccf-mtDNA fragmentomic features between mtDNA Cap-seq and WGS data. 5

Appendix Figure S5 | Consistency of ccf-mtDNA fragmentation profiles between mtDNA Cap-seq and WGS data from plasma samples of 30 healthy individuals. 6

Appendix Figure S6 | Influence of whole blood sample placement time on ccf-mtDNA fragmentation profiles. 7

Appendix Figure S7 | Influence of sequencing depth on ccf-mtDNA fragmentation profiles. 8

Appendix Figure S8 | Association of sex and age with fragmentation profiles of ccf-mtDNA.9

Appendix Figure S9 | Spearman's rank correlation coefficient between standardized mtDNA depth and FSD score 10

Appendix Figure S10 | Relationship of 5′ end base preference and motif diversity with fragment sizes of ccf-mtDNA from plasma samples of 30 healthy individuals. 11

Appendix Figure S11 | Different fragmentomic features of four functional regions of mtDNA from patients with different diseases. 13

Appendix Figure S12 | Fragmentomic features of ccf-mtDNA among HCs and patients with MT, BT and INF. 14

Appendix Figure S13 | The fragmentomic features of different ccf-mtDNA fragments. 16

Appendix Figure S14 | Establishment of cancer detection model (a) and tissue of origin classification model (b) based on fragmentomic features of ccf-mtDNA. 17

Appendix Figure S15 | Performance of cancer detection models in training cohort. 18

Appendix Figure S16 | Evaluation of cancer detection model among different cancer types in two validation cohorts. 19

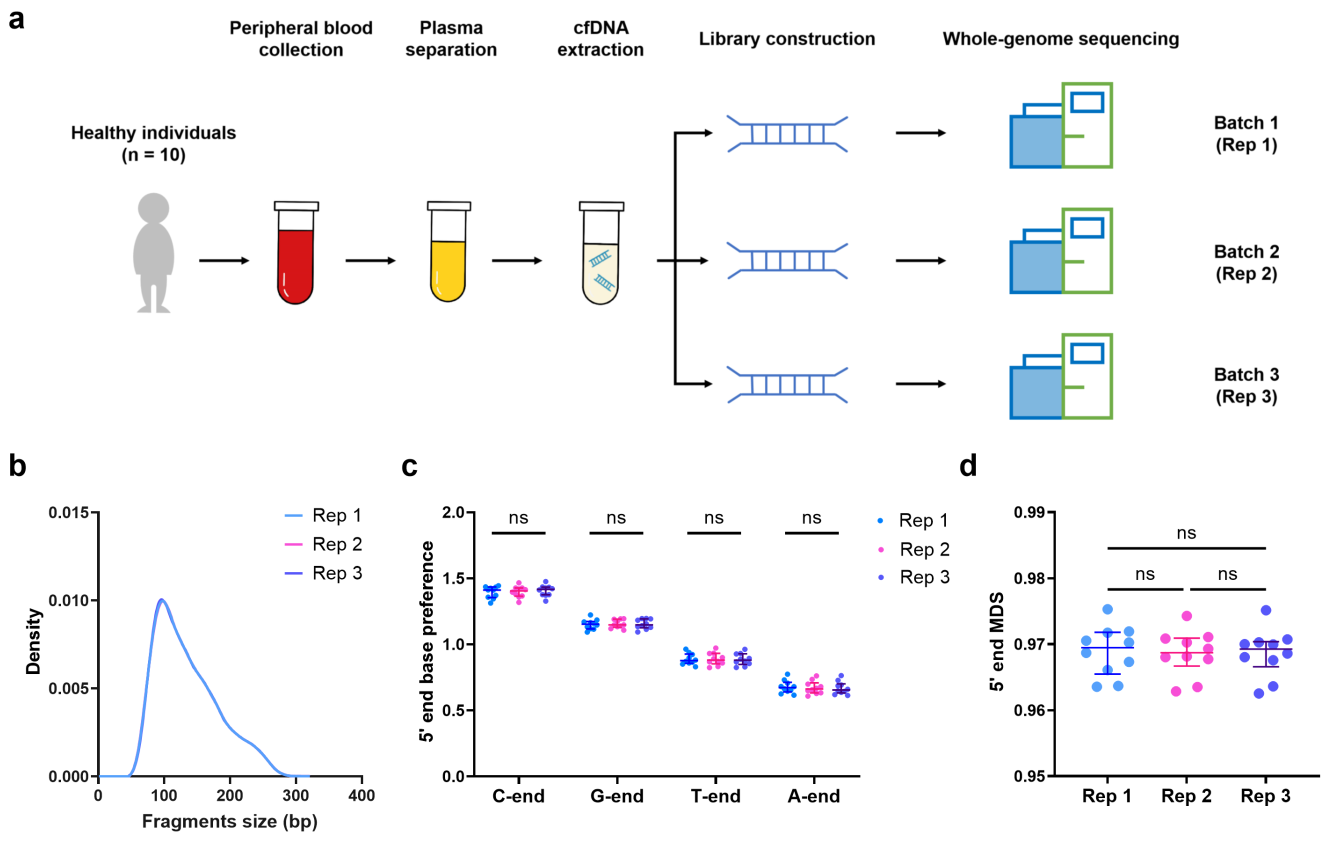

Appendix Figure S1 | Reproducibility of ccf-mtDNA fragmentomic features among three replicated experiments (Rep1-3). a. Workflow of three replicated experiments in 10 healthy individuals. **b-d.** Fragment size distribution **(b)**, 5' end base preference **(c)** and 5' end MDS **(d)** of ccf-mtDNA from plasma samples of 10 healthy individuals detected by WGS. Rep, replicate; MDS, motif diversity score; ns, not significant (Kruskal-Wallis H test and Dunn's multiple comparisons test). Center line indicates the median, lower and upper hinges represent the 25th and 75th percentiles, respectively.

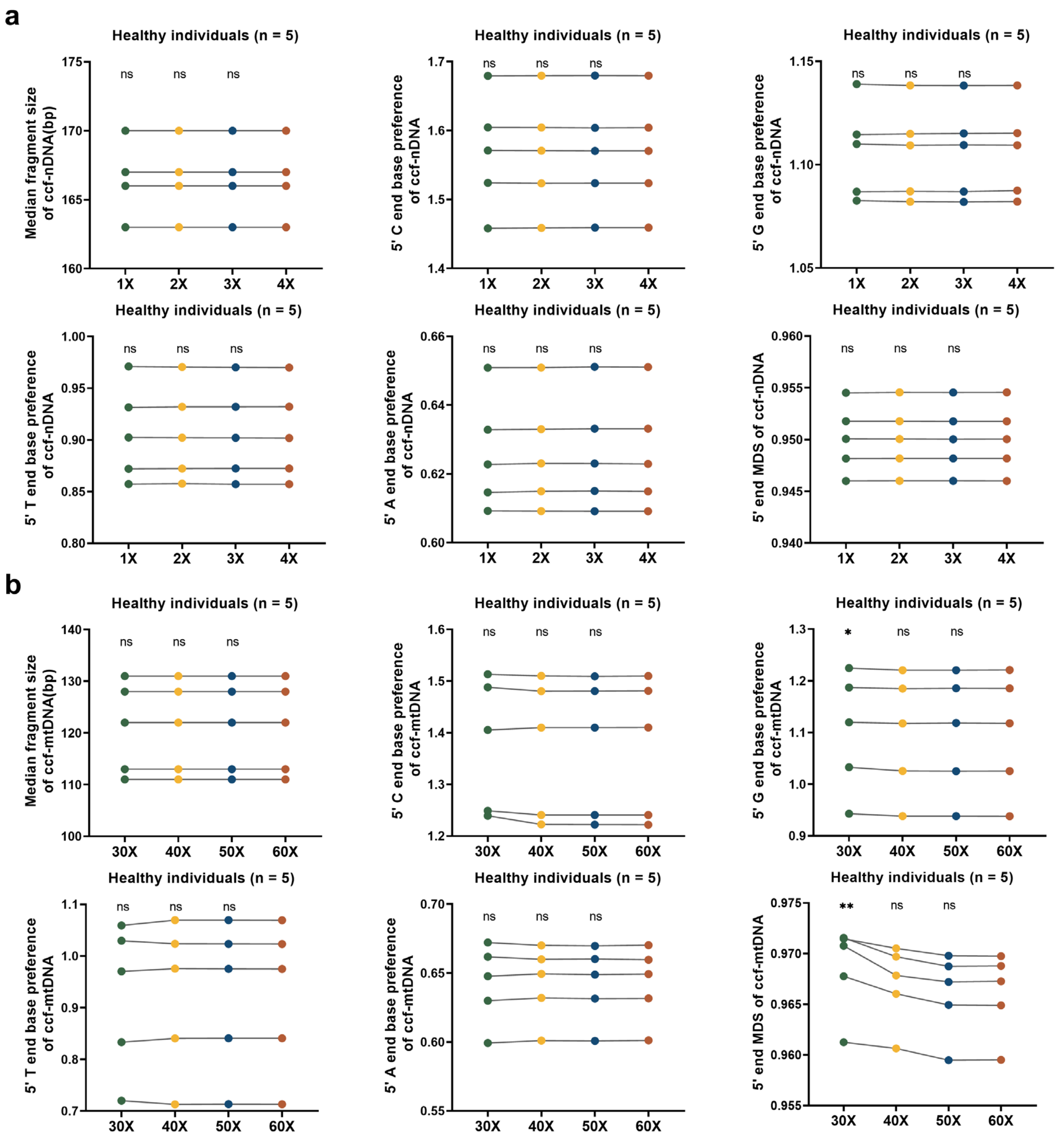
a
Healthy individuals (n = 5)
Median fragment size of ccf-nDNA(bp)
5' C end base preference of ccf-nDNA
5' G end base preference of ccf-nDNA
5' T end base preference of ccf-nDNA
5' A end base preference of ccf-nDNA
5' end MDS of ccf-nDNA
ns
1X
2X
3X
4X
b
Healthy individuals (n = 5)
Median fragment size of ccf-mtDNA(bp)
5' C end base preference of ccf-mtDNA
5' G end base preference of ccf-mtDNA
5' T end base preference of ccf-mtDNA
5' A end base preference of ccf-mtDNA
5' end MDS of ccf-mtDNA
ns
*
**
30X
40X
50X
60X

Appendix Figure S2 | Influence of sequencing depth on ccf-nDNA and ccf-mtDNA fragmentomic features. a. Median fragment size, 5’ end base preference and 5’ end MDS of 5 HCs detected by WGS data. Sequencing data with average coverage of 4 X were first extracted from each sample and then down sampled to 3 X, 2 X, 1 X. **b.** Median fragment size, 5’ end base preference and 5’ end MDS of 5 HCs detected by mtDNA Cap-seq data (*P* values: 5’ G end base preference of ccf-mtDNA: 0.0429, 5’ end MDS of ccf-mtDNA: 0.0099). Sequencing data with average coverage of 60 X were first extracted from each sample and then down sampled to 50 X, 40 X, 30 X. HC, healthy control; MDS, motif diversity score; ns, not significant; *$P < 0.05$; **$P < 0.01$; ***$P < 0.001$ (Friedman test).

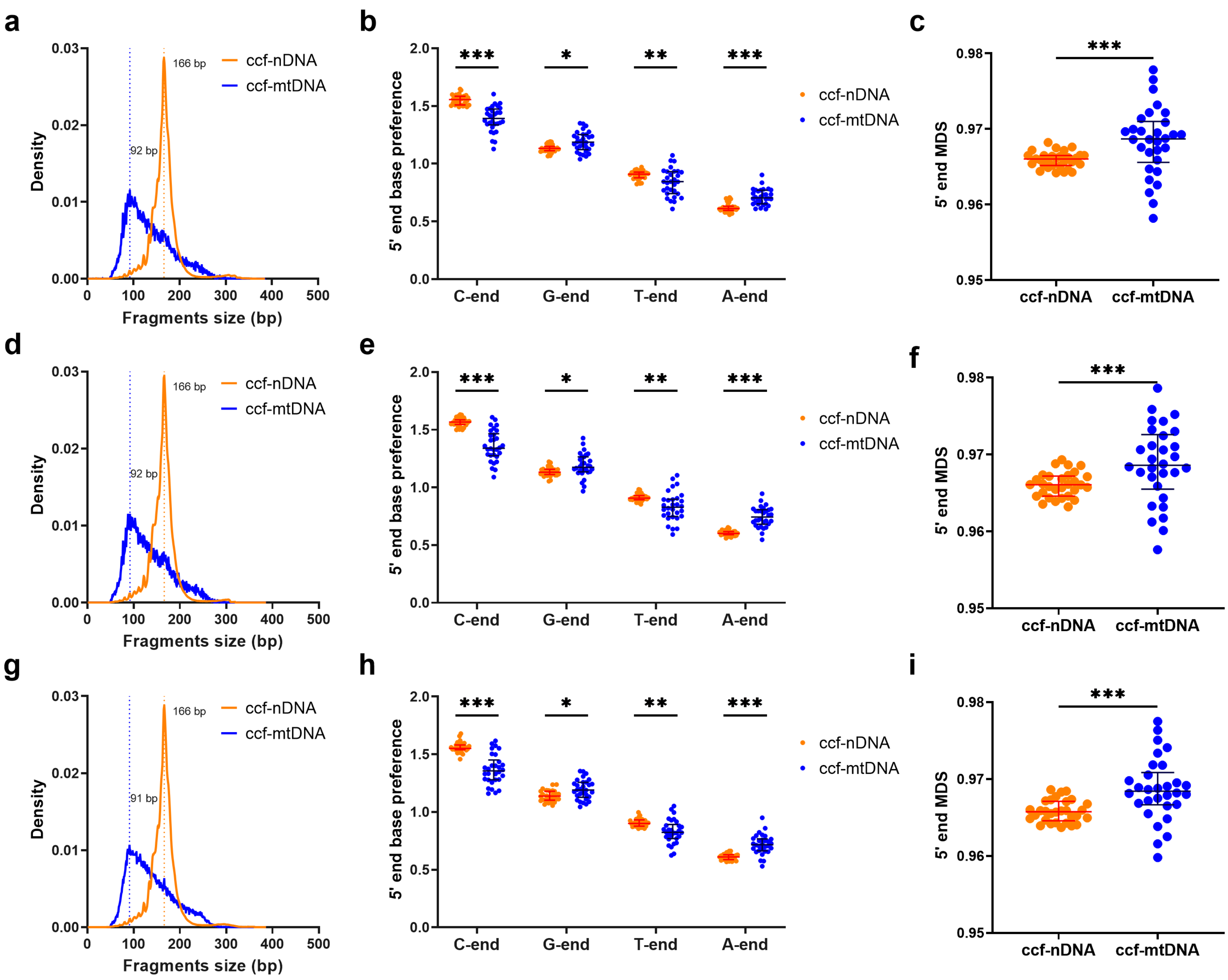

a
b
c
d
e
f
g
h
i
Density
Fragments size (bp)
166 bp
92 bp
91 bp
ccf-nDNA
ccf-mtDNA
5' end base preference
C-end
G-end
T-end
A-end
5' end MDS
***
*
**

Appendix Figure S3 | Different fragmentomic features between ccf-nDNA and ccf-mtDNA from patients with different disease types. a-i. Comparative analyses of fragment size distribution, 5'end base preference and 5' end MDS between ccf-nDNA and ccf-mtDNA from plasma samples of 30 patients with inflammatory diseases **(a-c)** (*P* values: b: < 0.0001; 0.0118; 0.0025; < 0.0001, c: 0.0003), 30 patients with benign tumor **(d-f)** (*P* values: e: < 0.0001; 0.0498; 0.0012; < 0.0001, f: 0.0005) and 30 patients with malignant tumor **(g-i)** (*P* values: h: < 0.0001; 0.0191; 0.0006; < 0.0001, i: < 0.0001) based on WGS data; MDS, motif diversity score; $*P < 0.05$; $**P < 0.01$; $***P < 0.001$ (Wilcoxon rank-sum test). Center line indicates the median, lower and upper hinges represent the 25th and 75th percentiles, respectively.

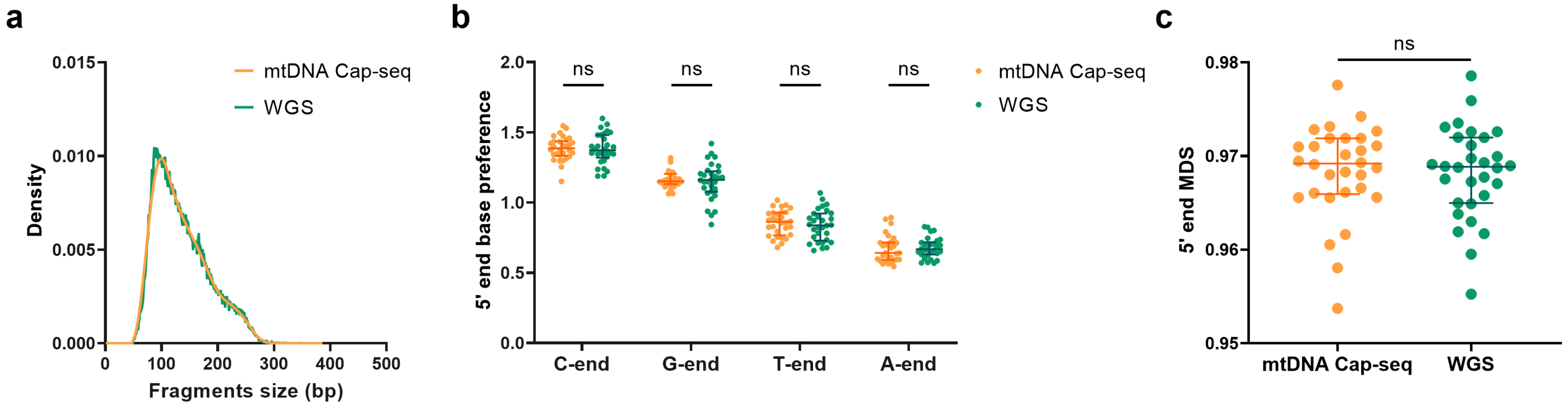

Appendix Figure S4 | Consistency of ccf-mtDNA fragmentomic features between mtDNA Cap-seq and WGS data. a-c. Fragment size distribution **(a)**, 5' end base preference **(b)** and 5' end MDS **(c)** of ccf-mtDNA in paired mtDNA Cap-seq and WGS data of plasma

samples from 30 healthy individuals. mtDNA Cap-seq, capture-based mtDNA sequencing; ns, not significant (Wilcoxon rank-sum test). Center line indicates the median, lower and upper hinges represent the 25th and 75th percentiles, respectively.

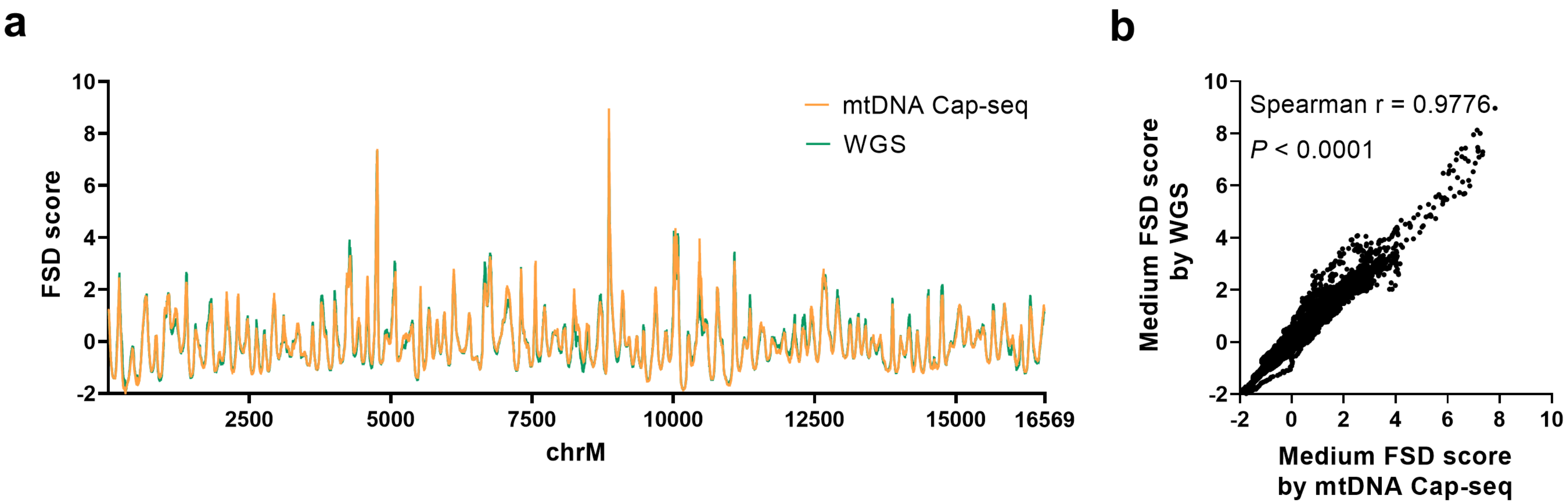

Appendix Figure S5 | Consistency of ccf-mtDNA fragmentation profiles between mtDNA Cap-seq and WGS data from plasma samples of 30 healthy individuals. A. Fragmentation profiles of ccf-mtDNA based on medium FSD score detected by mtDNA Cap-seq (orange) and WGS (green). **b.** Spearman's rank correlation coefficient of medium FSD score at each site between mtDNA Cap-seq and WGS data (*P* value: < 0.0001). mtDNA Cap-seq, capture-based mtDNA sequencing. Spearman's rank correlation coefficient was used to measure the associations between two groups.

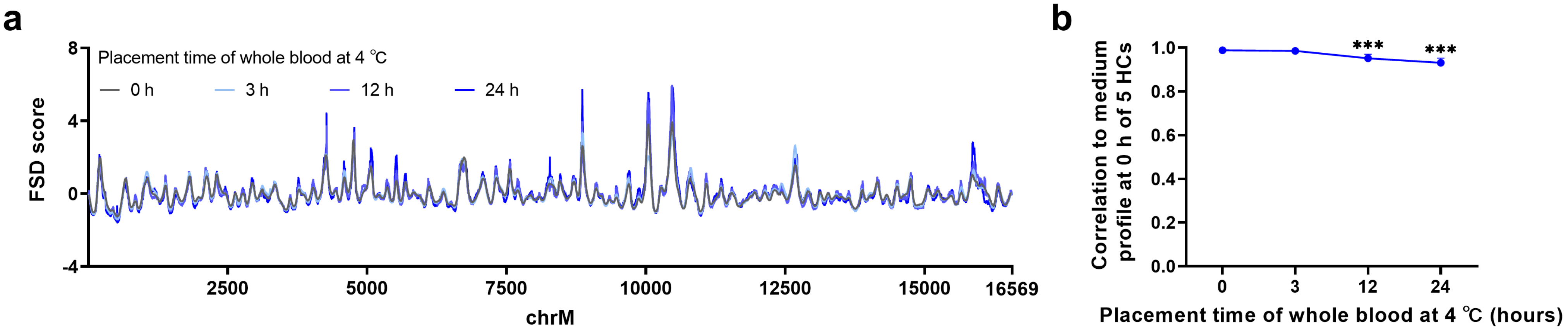

Appendix Figure S6 | Influence of whole blood sample placement time on ccf-mtDNA fragmentation profiles. a. Medium ccf-mtDNA fragmentation profiles of whole blood samples from 5 HCs at 4 °C. **b.** Spearman's rank correlation coefficient of fragmentation profiles of whole blood samples from 5 HCs at different placement times to medium fragmentation profile of 5 HCs at 0 h (*P* values: 0.0005; 0.0002). h, hour; HC, healthy control; ****P* < 0.001 (Wilcoxon rank-sum test).

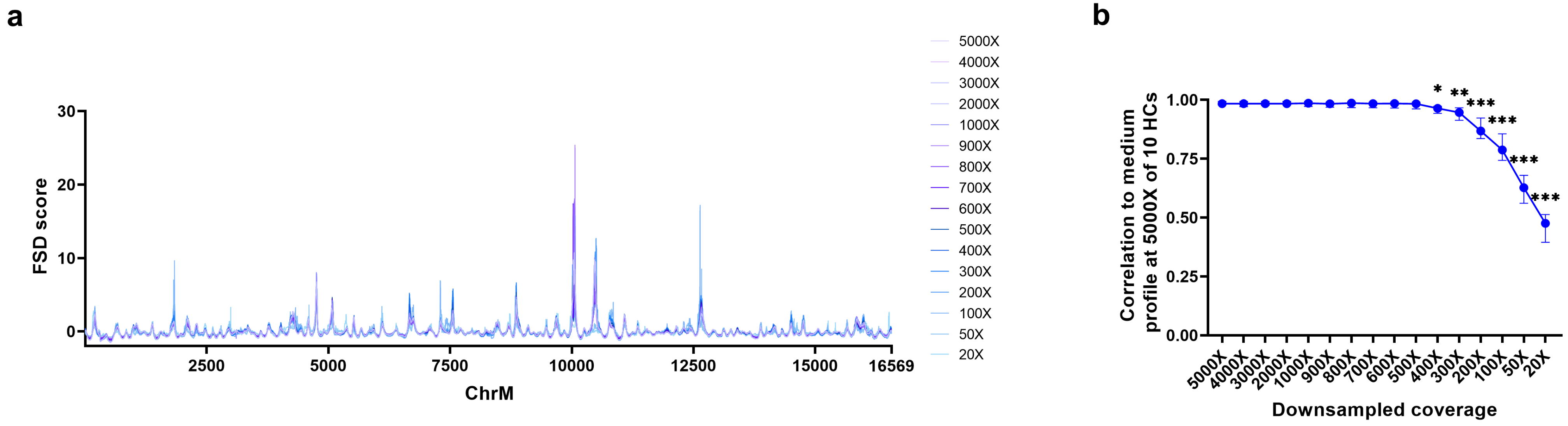

Appendix Figure S7 | Influence of sequencing depth on ccf-mtDNA fragmentation profiles. a. Medium ccf-mtDNA fragmentation profiles of 10 HCs detected by mtDNA Cap-seq data. Sequencing data with average coverage of 5000 X were first extracted from each sample and then down sampled to 4000 X, 3000 X, 2000 X, 1000 X, 900 X, 800 X, 700 X, 600 X, 500 X, 400 X, 300 X, 200 X, 100 X, 50 X, 20 X. **b.** Spearman's rank correlation coefficient of fragmentation profiles of 10 HCs at each down sampled coverage to medium fragmentation profile of 10 HCs at 5000 X (P values: 0.0101; 0.0052; 0.0009; 0.0003; < 0.0001; < 0.0001). HC, healthy control; *P < 0.05; **P < 0.01; ***P < 0.001 (Wilcoxon rank-sum test).

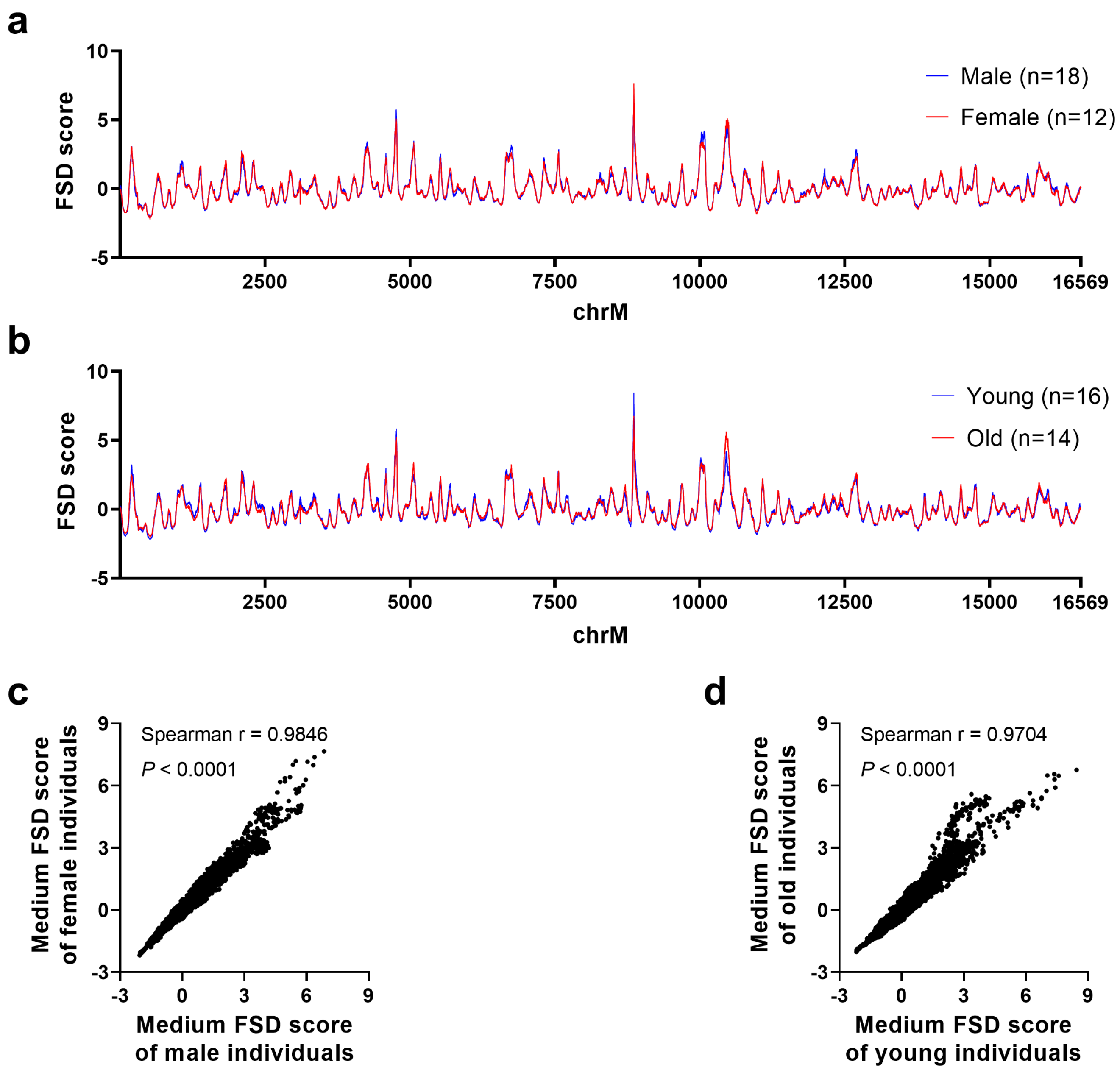

Appendix Figure S8 | Association of sex and age with fragmentation profiles of ccf-mtDNA. a. Medium ccf-mtDNA fragmentation profiles of male (n = 18) and female (n = 12) healthy individuals. **b.** Medium ccf-mtDNA fragmentation profiles of young (n = 16) and old (n = 14) healthy individuals stratified by medium age of 40 years. **c and d.** Spearman's rank correlation coefficient of medium FSD score at each site between male and female or between young and old healthy individuals (*P* values: c: < 0.0001, d: < 0.0001). Spearman's rank correlation coefficient was used to measure the associations between two groups.

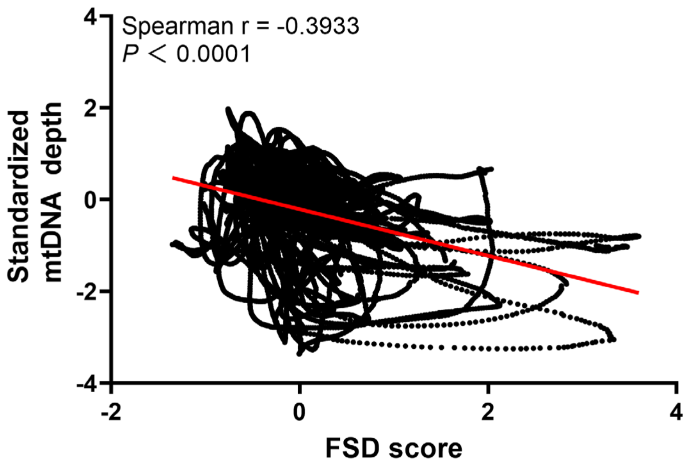

Appendix Figure S9 | Spearman's rank correlation coefficient between standardized mtDNA depth and FSD score. Standardized mtDNA depth was obtained from mitochondrial single cell ATAC sequencing data. FSD score was obtained from capture-based mtDNA sequencing data (*P* value: < 0.0001). FSD, fragment size distribution; mtscATAC-seq. Spearman's rank correlation coefficient was used to measure the associations between two groups.

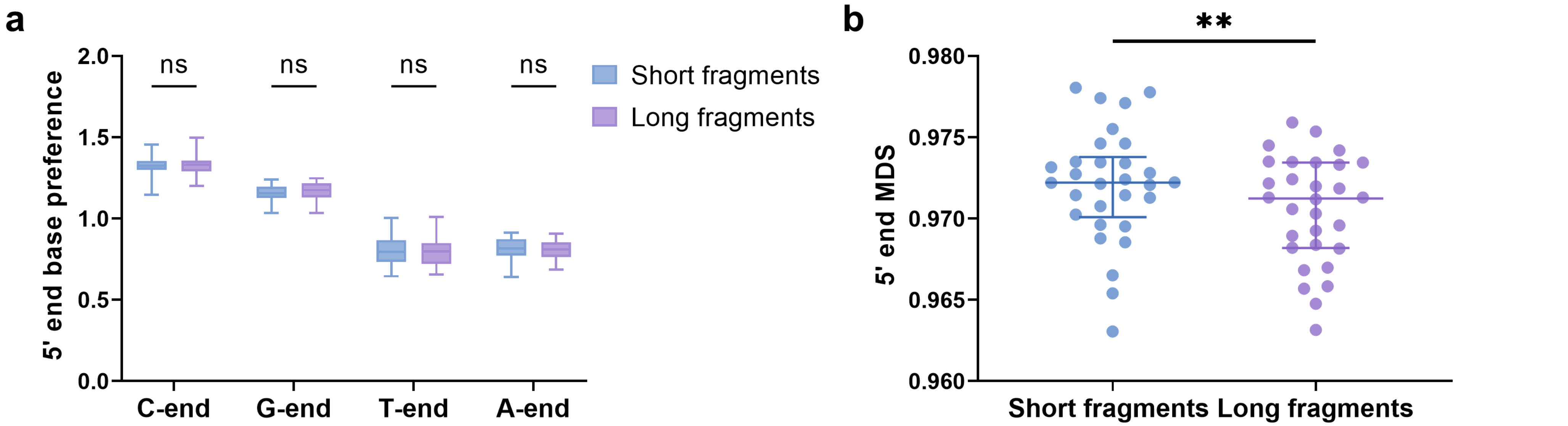

Appendix Figure S10 | Relationship of 5′ end base preference and motif diversity with fragment sizes of ccf-mtDNA from plasma samples of 30 healthy individuals. a and b. 5′ end base preference **(a)** and 5′ end MDS **(b)** (*P* value: 0.0011) of short and long ccf-mtDNA fragments stratified by the medium length; MDS, motif diversity score; ns, not significant; ***P* < 0.01 (Wilcoxon rank-sum test). In a, Boxes represent the 25th – 75th percentiles, center line indicates the median, whiskers extend to the maximum and minimum values within 1.5× interquartile range. In b, center line indicates the median, lower and upper hinges represent the 25th and 75th percentiles, respectively.

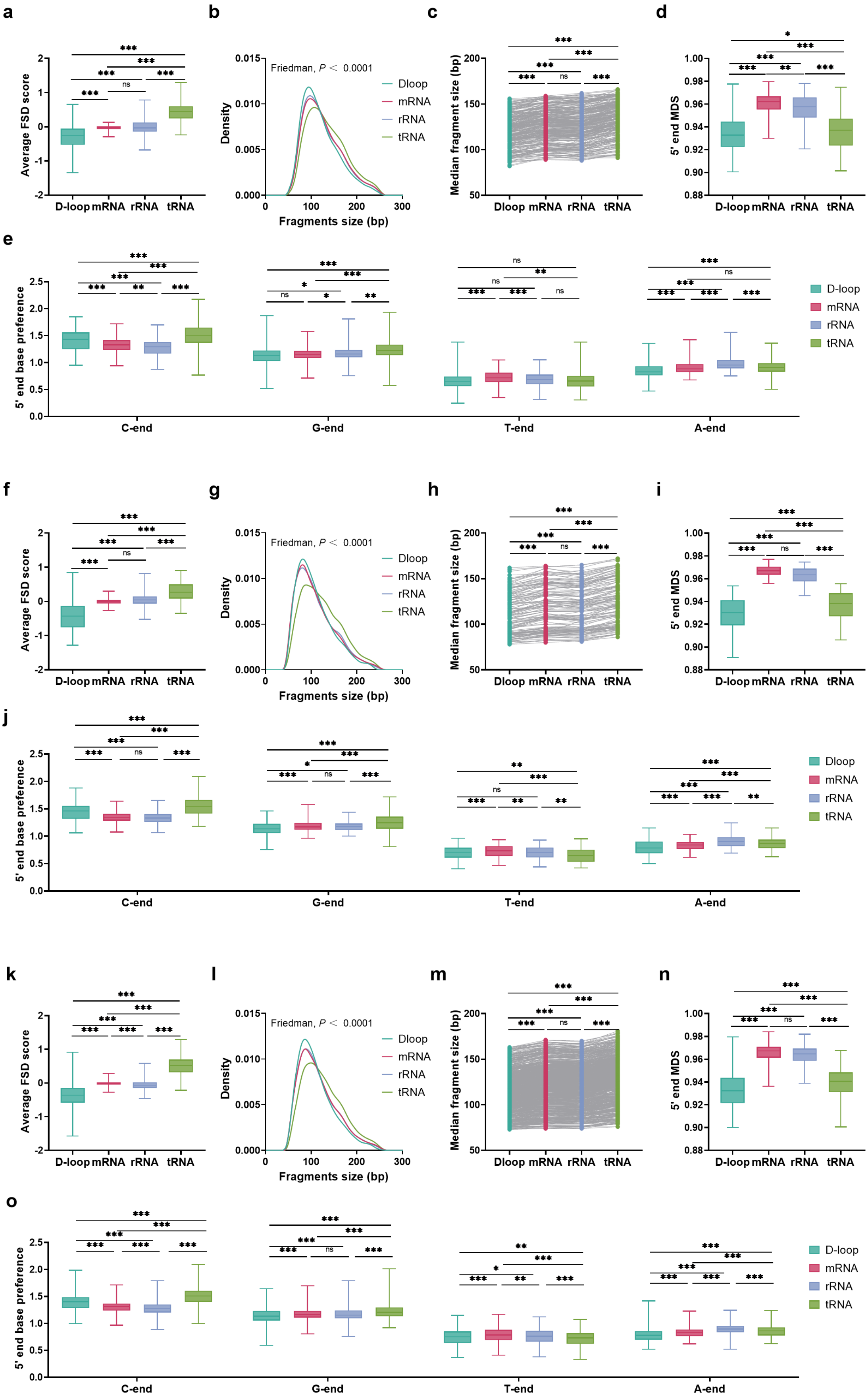

a
b
c
d
Average FSD score
D-loop mRNA rRNA tRNA
Density
Friedman, P < 0.0001
Dloop
mRNA
rRNA
tRNA
Fragments size (bp)
Median fragment size (bp)
Dloop mRNA rRNA tRNA
5' end MDS
e
5' end base preference
C-end
G-end
T-end
A-end
D-loop
mRNA
rRNA
tRNA
f
g
h
i
j
k
l
m
n
o

Appendix Figure S11 | Different fragmentomic features of four functional regions of mtDNA from patients with different diseases. a-o. Comparison of average FSD score, Density of fragments size, the median fragment size, 5′ end MDS and 5′end base preference of ccf-mtDNA among D-loop, mRNA, rRNA and tRNA regions based on mtDNA Cap-seq data from plasma samples of 290 patients with inflammatory diseases **(a-e)** (*P* values: a: < 0.0001; < 0.0001; < 0.0001; < 0.0001; < 0.0001, b: < 0.0001, c: < 0.0001; < 0.0001; < 0.0001; < 0.0001; < 0.0001, d: < 0.0001; < 0.0001; 0.0153; 0.0034; < 0.0001; < 0.0001, e: C-end: < 0.0001; < 0.0001; < 0.0001; 0.0089; < 0.0001; < 0.0001, G-end: 0.0440; < 0.0001; 0.0235; < 0.0001; 0.0022, T-end: 0.0007; < 0.0001; 0.0028, A-end: < 0.0001; < 0.0001; < 0.0001; < 0.0001; < 0.0001), 140 patients with benign tumor **(f-j)** (*P* values: f: < 0.0001; < 0.0001; < 0.0001; < 0.0001; < 0.0001, g: < 0.0001, h: < 0.0001; < 0.0001; < 0.0001; < 0.0001; < 0.0001, i: < 0.0001; < 0.0001; 0.0004; < 0.0001; < 0.0001, j: C-end: < 0.0001; < 0.0001; < 0.0001; < 0.0001; < 0.0001, G-end: 0.0009; 0.0350; < 0.0001; < 0.0001; < 0.0001, T-end: 0.0001; 0.0083; 0.0011; < 0.0001; 0.0034, A-end: 0.0010; < 0.0001; < 0.0001; < 0.0001; < 0.0001; 0.0051) and 877 patients with malignant tumor **(k-o)** (*P* values: k: < 0.0001; < 0.0001; < 0.0001; < 0.0001; < 0.0001; < 0.0001, l: < 0.0001, m: < 0.0001; < 0.0001; < 0.0001; < 0.0001; < 0.0001, n: < 0.0001; < 0.0001; < 0.0001; < 0.0001; < 0.0001, o: C-end: < 0.0001; < 0.0001; < 0.0001; 0.0002; < 0.0001; < 0.0001, G-end: < 0.0001; 0.0001; < 0.0001; < 0.0001; < 0.0001, T-end: < 0.0001; 0.0450; 0.0060; 0.0029; < 0.0001; < 0.0001, A-end: < 0.0001; < 0.0001; < 0.0001; < 0.0001; < 0.0001; < 0.0001). Average FSD score was calculated based on all sites in a given functional region; MDS, motif diversity score; ns, not significant; $*P < 0.05$; $**P < 0.01$; $***P < 0.001$ (Kruskal-Wallis H test and Dunn's multiple comparisons test). The Friedman test was used to compare the difference of

fragment size distribution. The center line indicates the median, lower and upper hinges represent the 25th and 75th percentiles, respectively. The boxes represent the 25th – 75th percentiles, center line indicates the median, whiskers extend to the maximum and minimum values within 1.5× interquartile range.

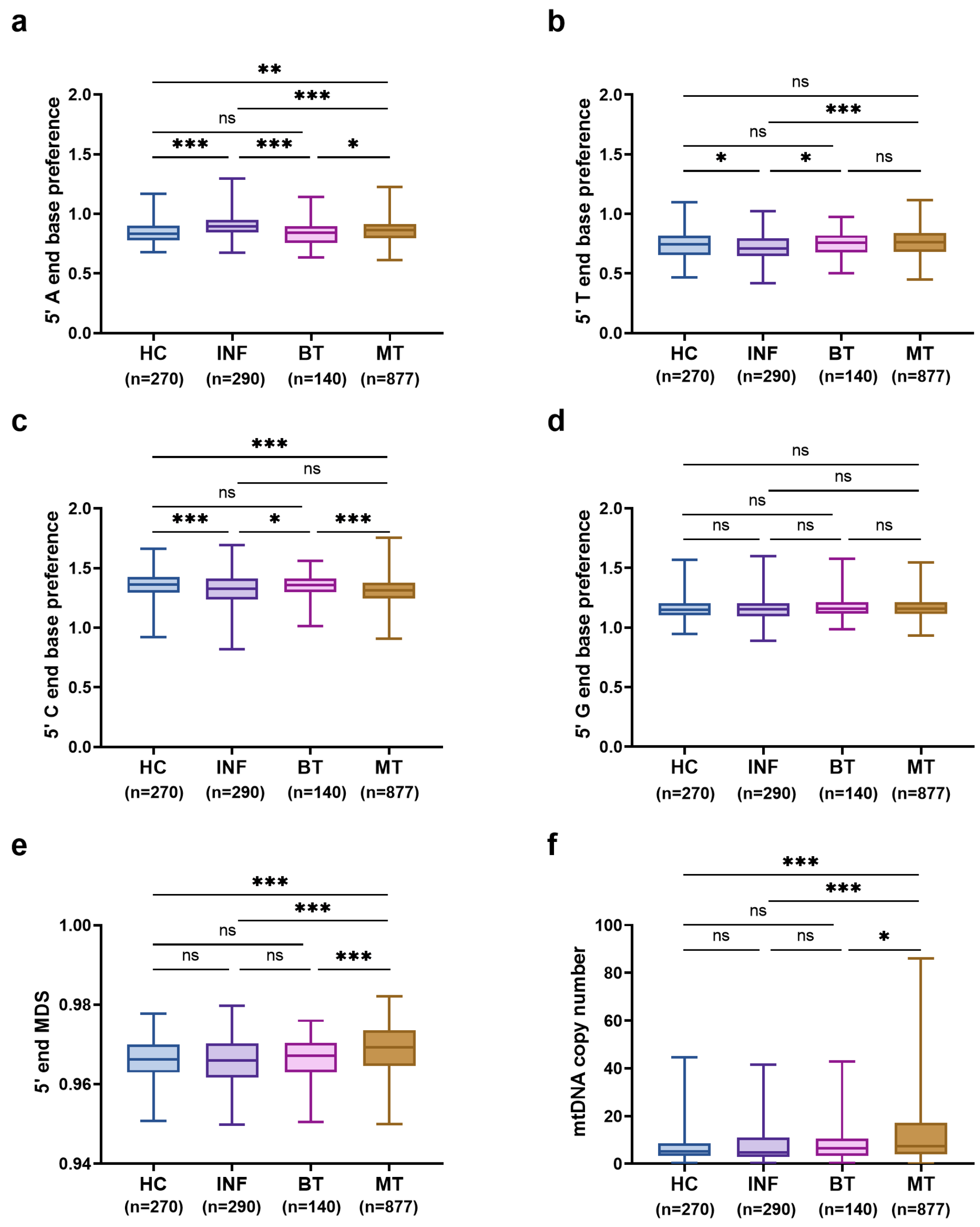

Appendix Figure S12 | Fragmentomic features of ccf-mtDNA among HCs and patients with MT, BT and INF. a-f. Comparison of 5′ end base preference **(a-d)** (*P* values: a: < 0.0001; 0.0076; < 0.0001; < 0.0001; 0.0146, b: 0.0389; 0.0133; < 0.0001, c: 0.0004; < 0.0001; 0.0409; < 0.0001), 5′ end MDS **(e)** (*P* values: < 0.0001; < 0.0001; 0.0005) and

mtDNA copy number **(f)** (*P* values: < 0.0001; < 0.0001; 0.0450) of ccf-mtDNA among four groups. HC, healthy control; MT, malignant tumor; BT, benign tumor; INF, inflammation; MDS, motif diversity score; ns, not significant; **P* < 0.05; ***P* < 0.01; ****P* < 0.001 (Kruskal-Wallis H test and Dunn's multiple comparisons test). Boxes represent the 25th – 75th percentiles, center line indicates the median, whiskers extend to the maximum and minimum values within 1.5× interquartile range.

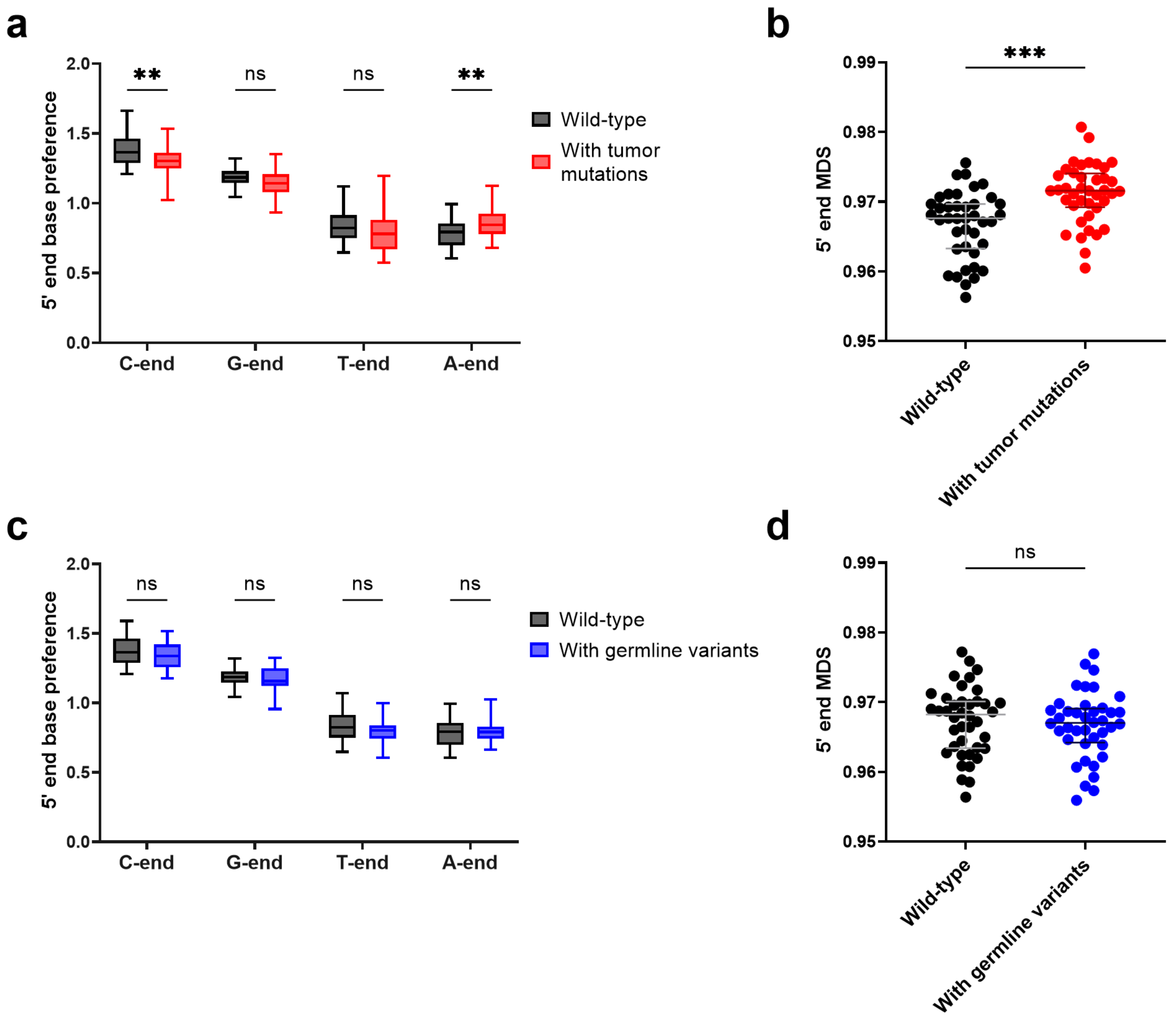

Appendix Figure S13 | The fragmentomic features of different ccf-mtDNA fragments. a-d. Comparison of 5′ end base preference and 5′ end MDS between fragments with tumor-derived mutations and corresponding wild-type ccf-mtDNA fragments **(a and b)** (*P* values: a: 0.0036; 0.0074, b: < 0.0001) and between fragments with germline variants and corresponding wild-type ccf-mtDNA fragments **(c and d)** from plasma samples of 40 patients with hepatocellular carcinoma; MDS, motif diversity score; ns, not significant; $^{**}P < 0.01$; $^{***}P < 0.001$ (Wilcoxon rank-sum test). In a and c, boxes represent the 25th – 75th percentiles, center line indicates the median, whiskers extend to the maximum and minimum values within 1.5× interquartile range. In b and d, center line indicates the median, lower and upper hinges represent the 25th and 75th percentiles, respectively.

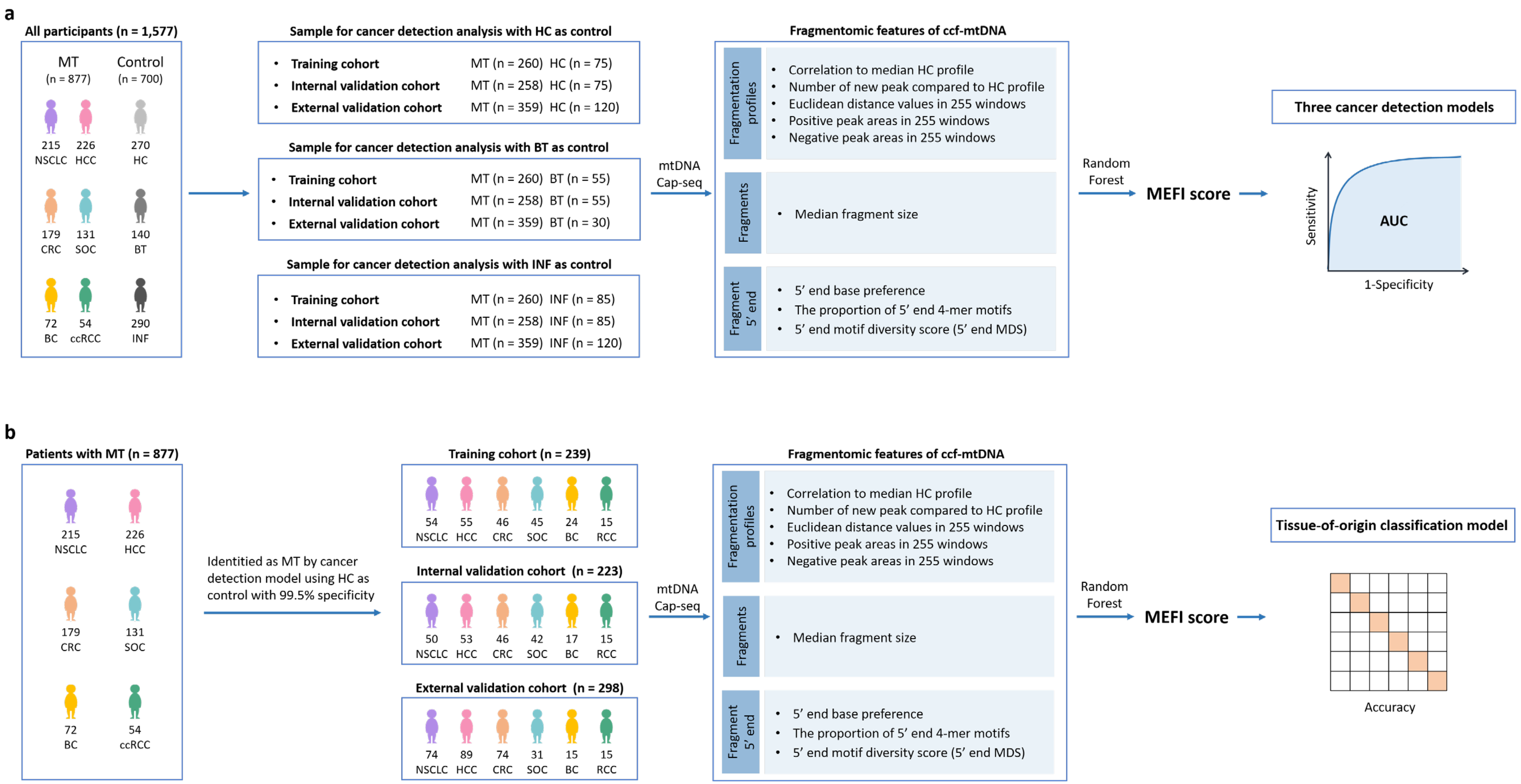

Appendix Figure S14 | Establishment of cancer detection model (a) and tissue of origin classification model (b) based on fragmentomic features of ccf-mtDNA. MEFI, mtDNA evaluation of fragmentomics for cancer investigation.

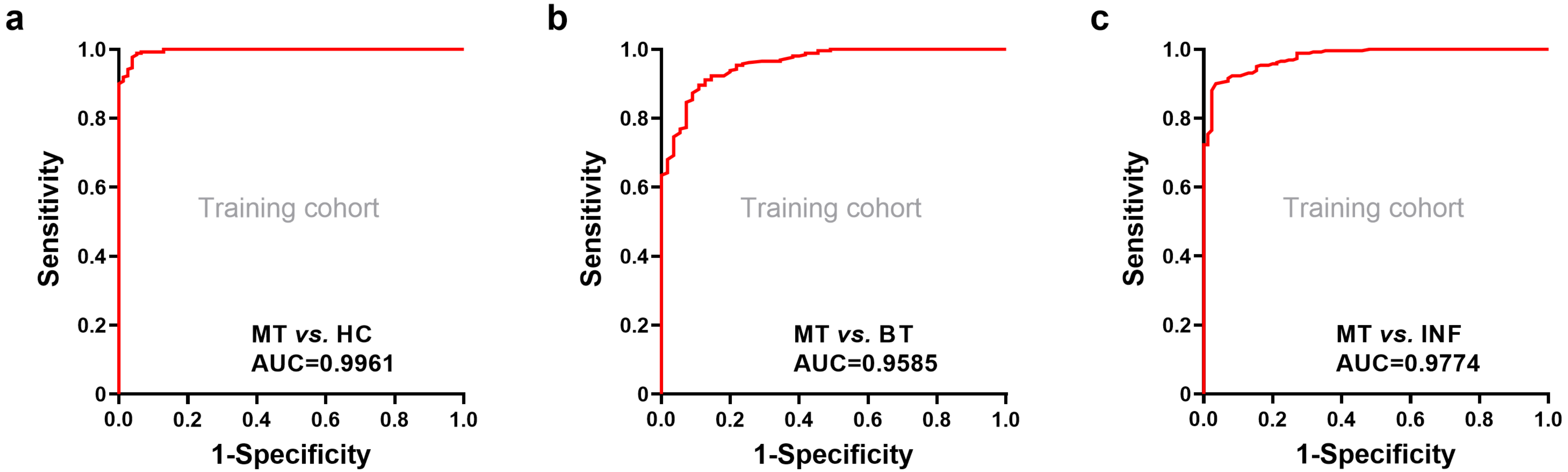

Appendix Figure S15 | Performance of cancer detection models in training cohort. a-c. Receiver operating characteristic curves evaluating the overall performance of three cancer detection models for distinguishing MT *vs*. HC **(a)**, MT *vs*. BT **(b)** and MT *vs*. INF **(c)** in training cohort. MEFI score was determined by tenfold cross-validation procedure using random forest algorithm. MT, malignant tumor; HC, healthy control; BT, benign tumor; INF, inflammation; AUC, area under the curve.

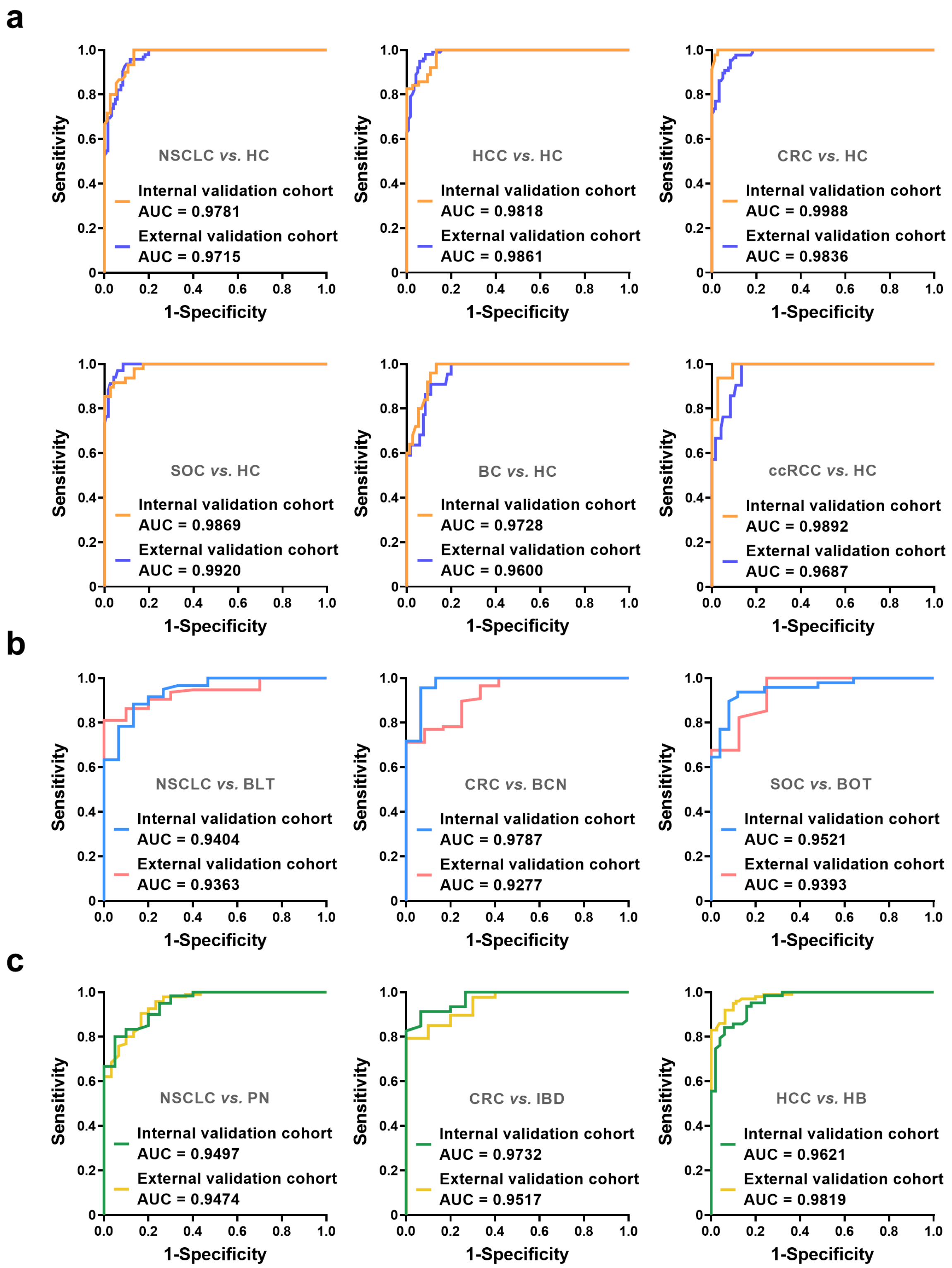

Appendix Figure S16 | Evaluation of cancer detection model among different cancer types in two validation cohorts. a. Receiver operating characteristic curves of MEFI score in detection of MT of 6 different types including non-small cell lung cancer (NSCLC), hepatocellular carcinoma (HCC), colorectal cancer (CRC), serous ovarian cancer (SOC),

breast cancer (BC), clear cell renal cell carcinoma (ccRCC) in the internal validation cohort and external validation cohort. **b and c.** Receiver operating characteristic curves of MEFI score in detecting different types of cancer when BT including benign lung tumors (BLT), benign colonic neoplasm (BCN), benign ovarian tumor (BOT) **(b)** and INF including pneumonia (PN), inflammatory bowel disease (IBD) and hepatitis B (HB) **(c)** were used as control in the internal validation cohort and external validation cohort. HC, healthy control; MT, malignant tumors; AUC, area under the curve.
